# Factors Affecting Hesitancy to mRNA and Viral Vector COVID-19 Vaccines among College Students in Italy

**DOI:** 10.3390/vaccines9080927

**Published:** 2021-08-19

**Authors:** Laura Salerno, Lucia Craxì, Emanuele Amodio, Gianluca Lo Coco

**Affiliations:** 1 Department of Psychology, Educational Science and Human Movement, University of Palermo, 90128 Palermo, Italy; laura.salerno@unipa.it (L.S.); gianluca.lococo@unipa.it (G.L.C.); 2 Department of Biomedicine, Neuroscience and Advanced Diagnostics, University of Palermo, 90127 Palermo, Italy; 3 Department of Health Promotion, Mother and Child Care, Internal Medicine and Medical Specialties, University of Palermo, 90127 Palermo, Italy; emanuele.amodio@unipa.it

**Keywords:** vaccine hesitancy, COVID-19, mRNA vaccines, viral vector vaccines, college students, Italy

## Abstract

Vaccine hesitancy (VH) may be significant in jeopardizing efforts to mass containment of COVID-19. A cross-sectional survey was carried out on a sample of 2667 Italian college students, before the COVID-19 vaccines became available for this age group (from 7 May to 31 May 2021). An online survey was created to obtain information about socio-demographic, health-related, and psychological factors linked to mRNA and viral vector COVID-19 vaccines. Statistically significant higher VH (30.4%) and vaccine resistance (12.2%) rates were found for viral vector than mRNA COVID-19 vaccines (7.2% and 1.0%, respectively; *p* < 0.001). Factors related to viral vector VH were partially different from those related to mRNA VH. Students with greater endorsement on conspiracy statements and negative attitudes toward the vaccine had higher odds of being vaccine-hesitant or -resistant. Students who had received a previous COVID-19 test and who scored higher on the agreeableness personality dimension had lower odds to be vaccine-hesitant or -resistant. The willingness to choose the vaccine was related to the viral vector but not to the mRNA VH. Taking into consideration the factors involved in vaccine hesitancy/resistance in college students could represent a key public health strategy to increase vaccine coverage and reduce viral spreading.

## 1. Introduction

There is substantial evidence that COVID-19 vaccines can effectively reduce transmission and that immunized individuals are likely to obtain protection against severe disease and death [1,2,3,4], even if it is not clear what uptake of vaccination is needed to achieve population immunity, given doubts about the spread of new COVID-19 variants and the duration of immunity. It is likely that vaccinating quickly and thoroughly can prevent new variants from gaining a foothold. Across countries, the vaccine distribution has been stratified by age and vulnerability, with priority given to the elderly who are at the highest risk of dying from COVID-19. However, adolescents and young adults also need to be immunized to achieve a “herd-immunity” threshold and reduce the total burden of COVID-19 disease. Thus, there has been a massive effort to improve vaccination rates and the efficiency of vaccine roll-outs among different age groups, in accordance with previous research on other vaccines which has shown population subgroups can react differently to the vaccine attributes [5].

A crucial concern is vaccine hesitancy, which could undermine efforts to control the pandemic [6] and poses a significant public health challenge. Vaccine hesitancy is defined as a delay in acceptance or refusal of vaccination despite availability of vaccination services [7]. It represents a complex phenomenon and is influenced by factors such as complacency (e.g., do not perceive a need for a vaccine), convenience (e.g., access), and confidence (e.g., lack of trust in vaccines) [8,9]. Although reports on the rate of COVID-19 vaccine hesitancy in 2020, when the vaccines were not available, raised some concerns and fluctuated with the waves of the pandemic [10,11,12,13], there is promising evidence of its reduction since vaccination programs rolled-out across the European region in 2021 [9,14].

Prior reviews on prevalence estimates of vaccine hesitancy showed that as the pandemic has progressed, the percentage of people intending to refuse vaccination increased [15], with 20% of people intending to refuse vaccination during June–October 2020. The review by Troiano and Nardi [8] also highlighted some factors that influenced the vaccine acceptance or refusal during March–November 2020, such as religiosity, political partisanship, being a woman, or lower level of education. Furthermore, there have been reports of widespread misinformation about COVID-19 and concerns about the safety of COVID-19 vaccines given their rapid development [16]. There is evidence that online rumors and conspiracy theories have had the potential to negatively impact the confidence of population towards the COVID-19 vaccines [17].

A relevant group of young adults to target for COVID-19 vaccination are college students, who are vulnerable to COVID infection due to factors such as their on-campus and off-campus housing, their broad social life, and their necessity to travel. Moreover, young people such as college students are usually healthy and often report mild symptoms after being infected with COVID-19. Thus, given their lower risk perception, they could show limited willingness for vaccination [18]. Prior research showed that the rate of young adults between 18–29 years reporting hesitancy to receive the COVID-19 vaccine is high, ranging from 47% to 60% across studies [19,20]. A survey with college students from a Southern USA University showed that 47.5% of participants reported hesitancy to receive the vaccine between February and March 2021 [19]. A previous study with Italian college students found that medical students, students with a higher level of concern about COVID-19, and students with high vulnerability to COVID-19 can have greater vaccination intention [21].

There are several individual difference variables that could make college students and young adults hesitant towards the vaccines, including mistrust in authorities and research (e.g., with regard to the rapid vaccine development, negative side effects, and other adverse event [22]), belief in conspiracy theories, and lack of trust in government [23]. Moreover, lower vaccine acceptance was detected in female and individuals younger in age [8,10,24,25].

In the current study, attitudes towards vaccination were assessed, in addition to demographics, clinical- and health-related indicators, and information about COVID-19. We also examined participants’ trust in medicine, given previous evidence regarding their positive impact on increased confidences in vaccines [9,26], as well as participants’ personality traits, in accordance with previous research which supported an association between the willingness to get vaccinated and some Big Five personality traits [27].

In sum, the understanding of factors that can contribute to COVID-19 vaccine hesitancy among college students is imperative in order to develop effective strategies to increase COVID-19 vaccination. However, none of the previous studies examined whether the rate of vaccine hesitancy among college students differed with respect to the vaccine typology. Although relevant authorities such as the European Medicines Agency and the WHO established that the benefits of COVID-19 vaccines far outweigh any possible risk [28,29], the safety of AstraZeneca vaccine has been concerned by mass media and social media as well [30]. It could be argued that the willingness to receive vaccination is lower with respect to the AstraZeneca vaccine, given the negative societal sentiment against it [31,32,33]. This issue seems especially relevant in the Italian context, given the repeated conflicting decisions about the use of the AstraZeneca vaccine during the first months of the Italian inoculation campaign. More specifically, after the European Union approved the use of the AstraZeneca vaccine in January, the Italian government recommended its use only for people under the age of 55. However, in March 2021, in accordance with other European countries, the Italian government briefly halted AstraZeneca inoculations over concerns of rare, severe thrombosis, mainly in young people. More than 20 countries stopped distributing that vaccine for a week or more. To our knowledge, only one experimental study in Germany [34] evidenced that the willingness to be vaccinated increased when the vaccine preferences (i.e., the BioNTech vaccine vs. the AstraZeneca vaccine) were taken into account. Moreover, when participants were asked which vaccine they would prefer (in February 2021), 46.2% of them favored the BioNTech vaccine, whereas only 2.3% chose the AstraZeneca vaccine, and 29.4% had no preferences.

In the current study, we conducted a survey of college students from a large Italian university, 5 months after the roll-out of the Italian vaccination program, when about 18% of the Italian general population had already received two doses of vaccine. We assessed willingness to be vaccinated for COVID-19 by asking participants their intentions for both the mRNA and viral vector vaccines. Moreover, we examined the association between vaccine hesitancy and socio-demographic characteristics, BIG Five personality traits, conspiracy beliefs, attitudes towards medicine, and sources of information about COVID-19.

## 2. Materials and Methods

### 2.1. Participants and Procedure

College students from the University of Palermo were invited, from 7 May to 31 May 2021, to participate in a cross-sectional online survey about vaccine hesitancy and some potential factors (i.e., socio-demographic, health-related, and psychological variables) which could be related to vaccine hesitancy. To be included in the study, participants should have not received the vaccine yet. In response to the call for participation, 3907 students volunteered to participate in this study. Participants who had already been vaccinated (*n* = 1240) were excluded from the analysis. In total, 2667 students were included in this study (age range: 18–56 years). Participants’ demographic and clinical information are reported in Table 1. The study was conducted in accordance with the ethical standards of the Italian Psychological Association (AIP), as well as the Declaration of Helsinki. It received the approval of the Ethics Committee of the University of Palermo. All participants completed statements of informed consent to participate in the study.

### 2.2. Measures

The online-structured self-administered questionnaire was anonymous and took approximately 15–20 min to be completed. Before starting the questionnaire, written information about the topic and the aim of the study was provided.

#### 2.2.1. Demographic and Clinical Data

The first part of the questionnaire was used to collect data about participants’ demographic information (i.e., sex, age, level of education, marital status, employment status, and degree course), clinical and health-related indicators (i.e., pregnancy and presence of pathologies within the family), and information about COVID-19 (i.e., students’ previous COVID-19 test results and diagnoses of COVID-19 among relatives).

#### 2.2.2. Vaccine Hesitancy

Vaccine hesitancy, separately for mRNA (i.e., Pfizer-BioNTech, Moderna, Curevac) and viral vector (i.e., AstraZeneca, Johnson & Johnson) COVID-19 vaccines, was evaluated with two questions (one for mRNA and one for viral vector COVID-19 vaccines, respectively) adapted from a previous study [27]. Participants were asked, “Would you receive a mRNA/viral vector COVID-19 vaccine when it becomes available for your age group?”. Participants were classified as “vaccine-accepting” if they responded “Certainly yes”, “vaccine-hesitant” if they responded “Maybe”, and “vaccine-resistant” if they responded “Certainly not”.

#### 2.2.3. Participants’ Willingness to Choose the Vaccine

Participants were asked, “Would you like to choose the type of COVID-19 vaccine to receive?” (1 = yes, 0 = no).

#### 2.2.4. Conspiracy Belief

The Vaccine Conspiracy Belief Scale [35] (VCBS) was used to measure conspiracy belief. The VCBS includes seven items (e.g., “Pharmaceutical companies cover up the dangers of vaccines”). Each item was rated on a seven-point Likert scale from 1 (Strongly disagree) to 7 (Strongly agree). Higher scores indicate greater endorsement of conspiracy statements. In the current study, the VCBS showed excellent reliability (Cronbach’s alpha value = 0.943).

#### 2.2.5. Attitudes towards Medicine

Attitudes towards medicine were evaluated through nine items from the Attitude to Doctors and Medicine Questionnaire by Freeman et al. [36]. The selected nine items covered the following domains: positive attitude towards medicine (four items, e.g., “Medicine is based on scientific principles”) and negative attitude towards medicine (five items, e.g., “Most tests and investigations are done routinely rather than for a particular purpose”). Items were rated on a six-point Likert scale from 1 (Strongly disagree) to 6 (Strongly agree). Higher scores indicate greater positive or negative attitudes toward medicine. In the current study, the internal reliability was questionable (Cronbach’s alpha values = 0.603 and 0.687 for positive and negative attitudes toward medicine subscale, respectively).

#### 2.2.6. Personality

The Big Five Inventory [37,38] (BFI-10) was used to measure personality traits. The BFI-10 includes ten items covering the following domains: agreeableness, conscientiousness, emotional stability, extroversion, and openness (two questions were related to each personality domain). Each item was rated on a five-point Likert scale from 1 (Strongly disagree) to 5 (Strongly agree). Higher scores indicate greater levels on a given personality trait (score range for each domain = 2–10).

#### 2.2.7. Sources of COVID-19 Vaccine Information

Participants were asked how often they refer to traditional media (e.g., television, newspapers, magazines), social media (e.g., Facebook, Twitter, Instagram), or health professionals in order to obtain information about COVID-19 vaccines. For each option, responses were recorded on a five-point Likert scale from 1 (Never) to 5 (Very often).

#### 2.2.8. Attitudes toward Vaccine

Attitudes around vaccine complacency and confidence, separately for mRNA (i.e., Pfizer-BioNTech, Moderna, Curevac) and viral vector (i.e., AstraZeneca, Johnson & Johnson) COVID-19 vaccines, were evaluated with seven questions adapted from the Oxford COVID-19 Vaccine Confidence and Complacency Scale [36]. The following domains were covered: collective importance of a COVID-19 vaccine (3 items), beliefs that the respondents may get COVID-19 and the vaccine will work (2 items), speed of vaccine development (1 item), and side effects (1 item). Each item was rated on a five-point Likert scale from 1 to 5, with item-specific response options [36]. Higher scores indicate a greater degree of negative attitudes (Cronbach’s alpha values = 0.740 and 0.845 for mRNA and viral vector vaccines, respectively).

### 2.3. Statistical Analyses

Statistical analysis was performed with R Statistical Software version 4.0.3, Vienna, Austria. Descriptive statistics for continuous (i.e., means and standard deviations) and qualitative variables (i.e., frequencies and percentages) were computed. Participants’ characteristics were compared using the chi-square test for categorical variables and ANOVA for continuous variables (followed by post hoc Bonferroni tests). Furthermore, adjusted odds ratios (ORs) and 95% confidence intervals (CIs) were calculated by multinomial regression model in order to examine factors associated with mRNA or viral vector COVID-19 vaccine hesitancy or resistance (ref. Acceptance). Only variables with a *p*-value of <0.10 on univariate analysis were subjected to multivariable analysis and non-significant variables in the multivariable model were removed by a backward stepwise approach. The level of significance chosen for all analyzes was 0.05 two-tailed.

## 3. Results

### 3.1. Prevalence of Vaccine Hesitancy for mRNA vs. Viral Vector COVID-19 Vaccines

**Figure 1:**
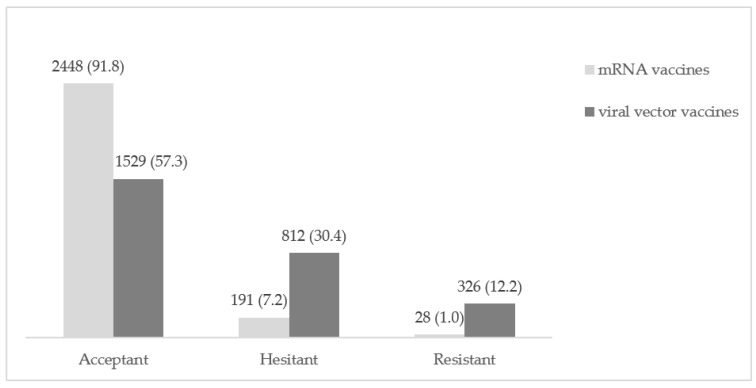
Rates of COVID-19 vaccine acceptance, hesitancy, and resistance for mRNA and viral vector vaccines separately; *n* (%).

Results about participants’ vaccine hesitancy are reported in Figure 1. Regarding mRNA COVID-19 vaccines, 91.8% (*n* = 2448) of participants were accepting of this type of vaccine, 7.2% (*n* = 191) were hesitant, and 1.0% (*n* = 28) were resistant.

Regarding viral vector COVID-19 vaccines, 57.3% (*n* = 1529) of participants were accepting of this type of vaccine, 30.4% (*n* = 812) were hesitant, and 12.2% (*n* = 326) were resistant.

The observed differences in the acceptance of the two vaccine types were statistically significant (*p* < 0.001).

The majority of the participants (*n* = 1647, 61.8%) rated consistently about the two vaccines. Out of participants who showed different opinion about the two types of vaccine (*n* = 1020, 38.2%), 1007 participants showed higher hesitancy about a viral vector vaccine.

### 3.2. Predictors of Vaccine Hesitancy for mRNA COVID-19 Vaccines

**Figure 2:**
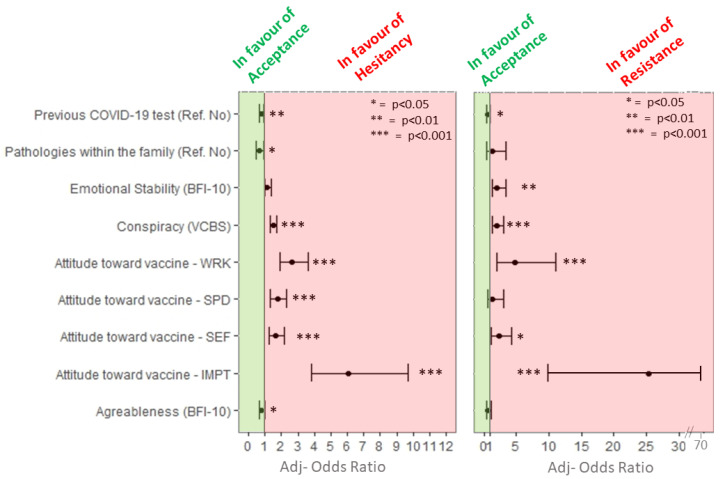
Multivariable analysis including variables statistically significantly associated with mRNA vaccine hesitancy. * *p* < 0.05; ** *p* < 0.01; *** *p* < 0.001.

Factors associated with mRNA COVID-19 vaccine hesitancy are presented in Table 2 and Figure 2. Univariate analyses showed an association between employment status (*p* < 0.01), presence of diseases among parents/relative/friends (*p* < 0.05), previous COVID-19 test (*p* < 0.001), and mRNA vaccine hesitancy. Moreover, hesitant and resistant students showed significantly higher conspiracy beliefs (*p* < 0.001), negative attitudes toward medicine (*p* < 0.001), negative attitudes toward vaccine (all subscales, *p* < 0.001), as well as significantly lower scores on agreeableness (*p* < 0.001), and positive attitudes toward medicine (*p* < 0.001) compared to acceptant participants. Regarding the sources of COVID-19 vaccine information, resistant students referred to traditional media lower than acceptant and hesitant participants (*p* < 0.001). In addition, hesitant students referred to health-professionals lower than acceptant participants (*p* < 0.01). No statistically significant differences were observed in mRNA COVID-19 vaccine hesitancy with regard to sex, educational level, degree course, marital status, diagnoses of COVID-19 among relatives, or conscientiousness, emotional stability, and openness to experience dimensions.

Data about multivariable regression model with mRNA COVID-19 vaccines hesitancy as dependent variable are reported in Figure 2. Students who had received previous COVID-19 tests had lower odds to be vaccine-hesitant (adj-OR 0.78, 95% CI: 0.65, 0.94) or vaccine-resistant (adj-OR 0.59, 95% CI: 0.35, 1) than students who did not get it. Students who reported the presence of pathologies within the family were less likely to be vaccine-hesitant (adj-OR 0.65, 95% CI: 0.46, 0.93).

Students with greater endorsement on conspiracy statements had higher odds of being vaccine-hesitant (adj-OR 1.51, 95% CI: 1.32, 1.73) or -resistant (adj-OR 1.99, 95% CI: 1.34, 2.97).

Regarding personality traits, students who scored higher on the agreeableness dimension were less likely to be vaccine-hesitant (adj-OR 0.78, 95% CI: 0.63, 0.97). In addition, students who scored higher on the emotional stability dimension were more likely to be vaccine-resistant (adj-OR 2.03, 95% CI: 1.23, 3.33).

Higher odds of being vaccine-hesitant or -resistant were related to negative attitudes around mRNA vaccines complacency and confidence. More specifically, the highest odds of being vaccine-hesitant or -resistant were seen in students with higher negative beliefs about the collective importance of the vaccine (hesitant = adj-OR 6.08, 95% CI: 3.81, 9.69; resistant = adj-OR 25.3, 95% CI: 9.8, 65.27) and with higher negative beliefs about the vaccine efficacy (hesitant = adj-OR 2.62, 95% CI: 1.91, 3.59; resistant = adj-OR 4.79, 95% CI: 2.07, 11.09). Moreover, students with higher negative beliefs about the side effects of the mRNA vaccines were more likely to be vaccine-hesitant (adj-OR 1.63, 95% CI: 1.24, 2.14) or -resistant (adj-OR 2.26, 95% CI: 1.19, 4.3). Finally, students with higher negative belief about the speed of vaccine development were more likely to be vaccine-hesitant (adj-OR 1.74, 95% CI: 1.32, 2.3). Table 3 summarizes factors associated with vaccine hesitancy.

### 3.3. Predictors of Vaccine Hesitancy for Viral Vector COVID-19 Vaccines

**Figure 3:**
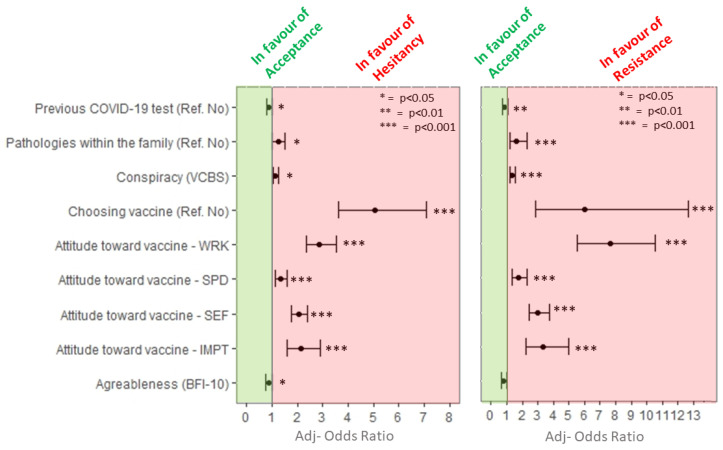
Multivariable analysis including variables statistically significantly associated with viral vector vaccine hesitancy. * *p* < 0.05; ** *p* < 0.01; *** *p* < 0.001.

Factors associated with viral vector COVID-19 vaccine hesitancy are presented in Table 2 and Figure 3. Univariate analyses showed an association between sex (*p* < 0.001), degree course (*p* < 0.001), marital status (*p* < 0.01), presence of diseases among parents/relatives/friends (*p* < 0.001), previous COVID-19 test (*p* < 0.001), and viral vector vaccines hesitancy. Moreover, hesitant and resistant students showed significantly higher conspiracy beliefs (*p* <0.001), negative attitudes toward medicine (*p* < 0.001), negative attitudes toward vaccines (all subscales, *p* < 0.001), as well as significantly lower scores on agreeableness (*p* < 0.001) and emotional stability (*p* < 0.001) dimensions, and positive attitudes toward medicine (*p* < 0.001) than acceptant participants. No statistically significant differences were observed in viral vector COVID-19 vaccine hesitancy with regard to educational level, occupation, diagnoses of COVID-19 among relatives, conscientiousness, extroversion and openness to experience dimensions, and sources of information.

Data about multivariable regression model with viral vector COVID-19 vaccines hesitancy as dependent variable are reported in Figure 3. Students who want to choose the type of COVID-19 vaccine to receive had higher odds of being vaccine-hesitant (adj-OR 5.06, 95% CI: 3.61, 7.1) and -resistant (adj-OR 6.03, 95% CI: 6.03, 95% CI: 2.87, 12.66).

Higher odds of being vaccine-hesitant or -resistant were related to negative attitudes around viral vector vaccines complacency and confidence. More specifically, higher odds of being vaccine-hesitant or -resistant were seen in students with higher negative beliefs about the vaccine efficacy (hesitant = adj-OR 2.87, 95% CI: 2.35, 3.52; resistant = adj-OR 7.64, 95% CI: 5.52, 10.57), with higher negative beliefs about the collective importance of the vaccine (hesitant = adj-OR 2.13, 95% CI: 1.57, 2.91; resistant = adj-OR 3.36, 95% CI: 2.26, 4.98), and with higher negative beliefs about the side effects of the mRNA vaccines (hesitant = adj-OR 2.05, 95% CI: 1.76, 2.38; resistant = adj-OR 3.01, 95% CI: 2.41, 3.77). Finally, students with higher negative beliefs about the speed of vaccine development were more likely to be vaccine-hesitant (adj-OR 1.35, 95% CI: 1.14, 1.59) or -resistant (adj-OR 1.76, 95% CI: 1.36, 2.29).

Students with greater endorsement on conspiracy statements had higher odds of being vaccine-hesitant (adj-OR 1.13, 95% CI: 1.02, 1.25) or -resistant (adj-OR 1.37, 95% CI: 1.19, 1.57).

Students who report the presence of pathologies within the family were more likely to be vaccine-hesitant (adj-OR 1.24, 95% CI: 1, 1.52) or -resistant (adj-OR 1.64, 95% Ci 1.17, 2.32).

Finally, regarding personality traits, students who scored higher on the agreeableness dimension were less likely to be vaccine-hesitant (adj-OR 0.87, 95% CI: 0.76, 0.99).

Table 3 summarizes factors associated with vaccine hesitancy.

## 4. Discussion

The present study was the first to separately examine the hesitancy for mRNA and viral vector COVID-19 vaccines among college students. Regarding the first aim of the study (i.e., to assess the willingness to be vaccinated for COVID-19), statistically significant differences were found between mRNA and viral vector COVID-19 vaccines. More specifically, vaccine hesitancy (30.4%) and resistance (12.2%) rates for the viral vector vaccines were higher than those reported for the mRNA COVID-19 vaccines (7.2% and 1.0%, respectively). Furthermore, whereas the majority of the participants rated consistently their willingness to vaccinate, slightly over a third of the participants showed higher hesitancy about the viral vector vaccine. Prior research has offered varying estimates of vaccine hesitancy across countries [6,8]. Our findings on vaccine hesitancy for the viral vector COVID-19 (42.6% for both VH and VR) vaccines substantially align with other estimates reported in previous studies among college students from European regions (Tavolacci et al. [39] reported that 42% of French university students were vaccine-hesitant or -resistant), and US (in the study by Sharma et al. [19], 47.5% of participants reported hesitancy to receive the COVID-19 vaccine), which assessed vaccine hesitancy without differentiating for the vaccine typology. Almost the same percentage was also found in Italian adult population [40] (a total of 42.0% for VH and VR) as well as in other European countries [41]. However, our findings about viral vector vaccine hesitancy/resistance are higher than those of Barello et al. [42] who reported 13.9% of Italian university students expressing they would not or be not sure to vaccine (low intention to be vaccinated). Moreover, our findings on vaccine hesitancy (8.2% for both VH and VR) for the mRNA COVID-19 vaccines were lower than those reported by previous studies [19,39], but aligned with the estimates reported by Graupensperger et al. [43] who found a higher percentage (91.64%) of college students from the US who reported intentions to get a COVID vaccine. The present study adds substantial evidence that the unwillingness to take a vaccine is not generic, but it is conditional on the specific vaccine typology, and could explain differences in VH rates in specific populations, such as college students and young adults. Most previous research on vaccine hesitancy is based on data which were collected in 2020 before the COVID-19 vaccines were authorized for public use, with surveys adopting generic wording about vaccination that provided no information about the different approved vaccines (i.e., mRNA and viral vector). Our findings suggest that the higher level of hesitancy for viral vectors vaccines can be related to a specific mistrust to them. In this sense, at least a part of these results could be due to misleading and sensationalized information reported by traditional and social media [30,32] and conflicting decisions during the Italian vaccination campaign.

Regarding the second aim of the study (i.e., to evaluate factors associated to vaccine hesitancy), our findings showed that multiple factors were associated to lower acceptance among college students, with some similarities and differences for the mRNA and viral vector COVID-19 vaccines. Regarding the demographic variables, none of the examined demographic variables showed significant associations with vaccine hesitancy (both mRNA and viral vector vaccines) in multivariate analyses. These findings are in line with those from the OCEAN survey in UK which suggested that socio-demographics did not explain vaccine hesitancy to a helpful degree [24]. Moreover, no significant differences in the intention to be vaccinated as regard to the university curricula (i.e., healthcare vs. non-healthcare curricula) were found in a previous study among Italian college students [40].

Regarding the health-related characteristics of vaccine-hesitant or -resistant participants, having a previous COVID-19 test was related to vaccine hesitancy for both mRNA and viral vector vaccines. More specifically, participants who have previously taken at least one COVID-test were less likely to be hesitant or resistant for both mRNA and viral vector vaccines. It could be hypothesized that this finding could be related to fear of COVID-19 and risk perception that are well-known reasons for getting vaccinated among students, as in the general adult population [23]. Furthermore, participants who have at least one family member with a clinical pathology were more unwilling to be vaccinated with a viral vector vaccine but were less likely to be hesitant for mRNA vaccines. Prior research suggested that different types of pandemic-related fears are likely to be linked to vaccine acceptance. For example, COVID-19-related anxiety and health-related fears were associated with a higher willingness to be vaccinated [25]. Our findings could reflect the fact that subjects with “vulnerable” family members may increase the protection level by getting a vaccine with higher efficacy as for the mRNA vaccines. Commonly, mRNA vaccines could be perceived as more safe, suitable, or effective for “vulnerable” people and for those surrounding them. These possible explanations cannot be assumed based on the findings of this study, but rather taken as suggestion requiring further studies.

Interestingly, the willingness to choose the vaccine was related to the viral vector but not to the mRNA vaccine hesitancy. Participants who affirmed their willingness to choose the vaccine were more likely to be hesitant or resistant for a viral vector COVID-19 vaccine. Moreover, the highest weight compared to the other factors considered in the model highlights the importance of this construct. These findings corroborate those by Sprengholz et al. [34] who found that willingness to be vaccinated increased when people were assigned to their preferred vaccine.

As regards to the psychological characteristics of COVID-19 vaccine-hesitant or -resistant participants, the findings of the study showed that conspiracy beliefs were related to vaccine hesitance both for mRNA and viral vector COVID-19 vaccines. More specifically, COVID-19 vaccine-hesitant and -resistant participants were more likely to hold higher conspiracy beliefs. These findings are in line with previous studies which showed a link between higher levels of vaccine hesitancy and conspiracy beliefs [17,24]. Regarding personality traits, participants with higher scores on the agreeableness dimension were less likely to be vaccine-hesitant for both mRNA and viral vector vaccines. Finally, contrary to expectation, participants with higher scores on the emotional stability dimension were more likely to be mRNA COVID-19 vaccine-resistant. The current findings partially support those from a large study among the UK and Ireland population [27] which showed that vaccine-hesitant or -resistant persons were distinguished from their vaccine-accepting counterparts by being more distrusting of experts, such as scientists and health care professionals, and more likely to hold strong religious beliefs as well as conspiratorial and paranoid beliefs. Moreover, they also had a personality characterized by being more disagreeable, more emotionally unstable, and less conscientious. Our findings suggest that vaccine-resistant individuals may display a tendency to high self-assurance and low reactance to stress and are firm in their willingness to avoid vaccination, given their concerns and negative beliefs about the vaccine efficacy, confidence, and complacency. On the contrary, participants who were resistant to viral vector vaccines showed average lower emotional stability than acceptant participants, even if this link between low emotional stability and vaccine resistance did not hold in multivariate analyses. It could be speculated that college students who reported a resistance to being vaccinated with viral vector vaccines may be influenced by the negative communication and societal sentiment against the AstraZeneca vaccine, given their tendency to show health anxiety and emotional reactance.

Regarding the attitudes in relation to vaccine complacency and confidence, higher negative attitudes were related to increased hesitance and resistance for both mRNA and viral vector COVID-19 vaccines. More specifically, COVID-19 vaccine-hesitant and -resistant participants were more likely to report greater negative concerns about side effects, the collective importance, and the low efficacy of a COVID-19 vaccine (both mRNA and viral vector vaccines). Finally, participants with higher negative attitudes towards the speed of vaccine development were more likely to be hesitant or resistant. These findings are in line with previous studies which showed that individuals with greater COVID-19 vaccine hesitancy have reported greater concerns about vaccine safety generally [44], and vaccine attributes posed prevalent concerns due to the novelty of COVID-19 vaccines [6]. A large survey in China [45] showed that 35.5% of participants in January 2021 reported vaccine hesitancy, and its most prevalent reason was concern about the safety of the COVID-19 vaccine and the low efficacy of the vaccine. However, there is evidence of substantial variations in COVID-19 vaccine acceptance between countries and subgroups, as well as in psychosocial predictors of vaccine hesitancy [6].

### Limitation of the Study

This study was subject to several potential limitations. Firstly, the study was limited by the cross-sectional design. Consequently, we do not know whether the psychological factors, beliefs, and attitudes towards the vaccines predict willingness to take a COVID-19 vaccine across the waves of the pandemic. It is likely that individuals’ attitudes towards COVID-19 vaccines will change with the different pandemic phases and further research is needed to monitor the vaccine confidence among college students. Secondly, participants of this study were recruited in one university in the south of Italy and caution is advised when generalizing these findings to different populations. Finally, further research is necessary to explore the role of political and religious beliefs to elucidate potential differences in uptake.

## 5. Conclusions

The findings of the current study suggest that students’ vaccine hesitancy is higher for viral vector than mRNA COVID-19 vaccines. These results offer a snapshot of young people’s perceptions in Italian COVID-19 pandemic experience after the vaccine roll-out, with millions of people who have been vaccinated. Moreover, this study suggests that individual willingness to be vaccinated is not influenced by socio-demographic characteristics; rather, health-related and psychological factors play as behavioral drivers. At this stage of the vaccination campaign, identifying factors associated with vaccine hesitancy and resistance among young adults is an important research effort, in order to inform community-led communication strategies (such as educational campaigns, research studies, and multidisciplinary approaches which involve peers, students’ organizations, or community leaders) to optimize COVID-19 vaccine uptake. Tailored educational interventions for college students aimed to improve their attitudes towards COVID-19 vaccines and avoid misinformation should be implemented [39], alongside public health campaigns. These education messages should also take into account differences in rate of vaccine hesitancy with respect to the vaccine type.

## Figures and Tables

**Table 1:** Demographics and health-related data.

	Total Sample *n* = 2667
Sex, *n* (%) females	1817 (68.1)
Age, M (SD)	22.74 (3.81)
Educational level, *n* (%)	
13 years of school	2036 (76.3)
Degree/post-degree	631 (23.7)
Marital status, *n* (%)	
Single/divorced/widowed	1280 (48.0)
Married/in a relationship	1387 (52.0)
Employment status, *n* (%)	
Unemployed	2316 (86.8)
Employed (part-time/full-time)	351 (13.2)
Diseases among parents/relatives/friends, *n* (%) yes	1530 (57.4)
Pregnancy, *n* (%) yes	4 (0.1)
Previous COVID-19 test, *n* (%)	
No test	992 (37.2)
Yes, at least one test	1675 (62.8)
Degree course, *n* (%)	
SH ^1^	1373 (51.5)
PE ^1^	757 (28.4)
LS ^1^	441 (16.5)
Missing	96 (3.6)
COVID-19 among relatives, *n* (%) yes	816 (30.6)
Willingness to choose the vaccine, *n* (%) yes	2089 (78.3)

^1^ SH: Social Sciences and Humanities; PE: Physical Sciences and Engineering; LS: Life Sciences.

**Table 2:** Variables associated with mRNA and viral vector COVID-19 vaccine hesitancy, separately.

	mRNA COVID-19 Vaccines				Viral Vector COVID-19 Vaccines			
	Acceptant	Hesitant	Resistant		Acceptant	Hesitant	Resistant	
	*n* (*%*)/M (SD)	*n* (*%*)/M (SD)	*n* (*%*)/M (SD)	*p*-Value	*n* (*%*)/M (SD)	*n* (*%*)/M (SD)	*n* (*%*)/M (SD)	*p*-Value
Total	2448 (91.8)	191 (7.2)	28 (1.0)		1529 (57.3)	812 (30.4)	326 (12.2)	
Sex								
Male	783 (92.1)	59 (6.9)	8 (0.9)	0.887	570 (67.1)	212 (24.9)	68 (8.0)	<0.001
Female	1665 (91.6)	132 (7.3)	20 (1.1)		959 (52.8)	600 (33.0)	258 (14.2)	
Educational level								
13 years of school	1868 (91.7)	149 (7.3)	19 (0.9)	0.494	1160 (57.0)	628 (30.8)	248 (12.2)	0.722
Degree/post-degree	580 (91.9)	42 (6.7)	9 (1.4)		369 (58.5)	184 (29.2)	78 (12.4)	
Degree course (*n* = 2571)								
SH ^1^	1257 (91.6)	100 (7.3)	16 (1.2)	0.778	734 (53.5)	448 (32.6)	191 (13.9)	<0.001
PE ^1^	699 (92.3)	52 (6.9)	6 (0.8)		481 (63.5)	210 (27.7)	66 (8.7)	
LS ^1^	411 (93.2)	26 (5.9)	4 (0.9)		271 (61.5)	119 (27.0)	51 (11.6)	
Marital status								
Single/divorced/widowed	1179 (92.1)	90 (7.0)	11 (0.9)	0.626	768 (60.0)	378 (29.5)	134 (10.5)	<0.01
Married/in a relationship	1269 (91.5)	101 (7.3)	17 (1.2)		761 (54.9)	434 (31.3)	192 (13.8)	
Employment status								
Unemployed	2141 (92.4)	154 (6.6)	21 (0.9)	<0.01	1340 (57.9)	706 (30.5)	270 (11.7)	0.065
Employed (Part-time/Full-time)	307 (87.5)	37 (10.5)	7 (2.0)		189 (53.8)	106 (30.2)	56 (16.0)	
Disease among parents/relatives/friends								
No	1027 (90.3)	100 (8.8)	10 (0.9)	<0.05	711 (62.5)	316 (27.8)	110 (9.7)	<0.001
Yes	1421 (92.9)	91 (5.9)	18 (1.2)		818 (53.5)	496 (32.4)	216 (14.1)	
Previous COVID-19 test								
No test	884 (89.1)	92 (9.3)	16 (1.6)	<0.001	524 (52.8)	330 (33.3)	138 (13.9)	<0.01
Yes, at least one test	1564 (93.4)	99 (5.9)	12 (0.7)		1005 (60.0)	482 (28.8)	188 (11.2)	
COVID-19 among relatives								
No	1701 (91.9)	130 (7.0)	20 (1.1)	0.894	1066 (57.6)	560 (30.3)	225 (12.2)	0.920
Yes	747 (91.5)	61 (7.5)	8 (1.0)		463 (56.7)	252 (30.9)	101 (12.4)	
Conspiracy	1.94 (1.11)	3.28 (1.40)	4.47 (1.19)	<0.001 ^2^	1.74 (.98)	2.29 (1.16)	3.08 (1.59)	<0.001 ^2^
Personality traits—BFI 10								
Agreeableness	3.26 (0.80)	3.06 (0.77)	2.84 (1.03)	<0.001 ^3^	3.29 (0.79)	3.20 (79)	3.10 (0.84)	<0.001 ^3^
Conscientiousness	3.63 (0.79)	3.67 (0.79)	3.75 (0.70)	0.592	3.61 (0.79)	3.65 (0.79)	3.68 (0.78)	0.332
Emotional Stability	2.73 (1.04)	2.77 (1.09)	3.16 (1.28)	0.083	2.81 (1.06)	2.63 (1.01)	2.64 (1.08)	<0.001 ^3^
Extroversion	2.93 (0.88)	2.77 (0.85)	3.09 (1.15)	0.036	2.95 (0.90)	2.86 (0.87)	2.89 (0.85)	0.052
Openness to experience	3.58 (0.89)	3.44 (0.93)	3.62 (0.92)	0.141	3.58 (0.89)	3.58 (0.87)	3.47 (0.96)	0.139
Attitude toward medicine								
Positive	19.28 (2.59)	17.84 (3.02)	16.82 (2.88)	<0.001 ^3^	19.40 (2.54)	18.97 (2.61)	18.40 (3.14)	<0.001 ^4^
Negative	15.14 (4.04)	17.14 (3.81)	18.11 (4.51)	<0.001 ^5^	14.70 (4.00)	15.82 (3.95)	16.90 (4.13)	<0.001 ^2^
Attitude toward vaccine								
Attitude toward vaccine—WRK ^8^	1.69 (0.58)	2.43 (0.66)	3.20 (0.88)	<0.001 ^2^	1.78 (0.57)	2.41 (0.61)	3.09 (0.77)	<0.001 ^2^
Attitude toward vaccine—SPD ^8^	2.36 (0.71)	3.03 (0.77)	3.68 (0.86)	<0.001 ^2^	2.33 (0.69)	2.77 (0.68)	3.33 (0.86)	<0.001 ^2^
Attitude toward vaccine—SEF ^8^	2.30 (0.56)	2.84 (0.84)	3.46 (1.07)	<0.001 ^2^	2.26 (0.55)	2.38 (0.61)	2.68 (0.79)	<0.001 ^2^
Attitude toward vaccine—IMPT ^8^	1.40 (0.26)	1.85 (0.52)	2.71 (.82)	<0.001 ^2^	1.43 (0.30)	1.70 (0.49)	2.26 (0.80)	<0.001 ^2^
Sources of information								
Traditional media	3.72 (1.01)	3.57 (1.01)	2.96 (1.11)	<0.001 ^6^	3.67 (1.03)	3.75 (0.96)	3.71 (1.05)	0.176
Social media	3.54 (1.18)	3.33 (1.21)	3.11 (1.29)	0.012	3.49 (1.19)	3.57 (1.14)	3.52 (1.25)	0.324
Medical health professionals	2.89 (1.16)	2.58 (1.12)	2.57 (1.17)	0.001 ^7^	2.90 (1.16)	2.81 (1.14)	2.85 (1.17)	0.184

^1^ SH: Social Sciences and Humanities; PE: Physical Sciences and Engineering; LS: Life Sciences; ^2^ resistant > hesitant > acceptant; ^3^ acceptant > hesitant, resistant; ^4^ acceptant > hesitant > resistant; ^5^ hesitant, resistant > acceptant; ^6^ acceptant, hesitant > resistant; ^7^ acceptant > hesitant; ^8^ WRK: efficacy of vaccine; SPD: speed of vaccine development; SEF: side effects; IMPT: collective importance of vaccine.

**Table 3:** Factors associated with mRNA and viral vector vaccines hesitancy.

mRNA Vaccine	Viral Vector Vaccine
***More Likelihood to be Hesitant/Resistant***	
Conspiracy beliefs	Willingness to choose the vaccine
Emotional Stability ^2^	Presence of pathologies within the family
Attitude toward vaccine—SPD ^1^	Conspiracy beliefs
Attitude toward vaccine—SEF	Attitude toward vaccine—SPD
Attitude toward vaccine—IMPT	Attitude toward vaccine—SEF
Attitude toward vaccine—WRK	Attitude toward vaccine—IMPT
	Attitude toward vaccine—WRK
***Less Likelihood to be Hesitant/Resistant***	
At least one previous COVID-19 test	At least one previous COVID-19 test ^1^
Presence of pathologies within the family ^1^	Agreeableness ^1^
Agreeableness ^1^	

^1^ Only for vaccine hesitance; ^2^ Only for vaccine resistance.

## Data Availability

Data are available by emailing the corresponding author.
